# Multistep, effective drug distribution within solid tumors

**DOI:** 10.18632/oncotarget.5051

**Published:** 2015-09-22

**Authors:** Amotz Shemi, Elina Zorde Khvalevsky, Rachel Malka Gabai, Abraham Domb, Yechezkel Barenholz

**Affiliations:** ^1^ Silenseed Ltd., Jerusalem, Israel; ^2^ Faculty of Medicine - School of Pharmacy, Hebrew University of Jerusalem, Jerusalem, Israel; ^3^ Membrane and Liposome Research Lab, Hebrew University Hadassah Medical School, Jerusalem, Israel

**Keywords:** drug delivery, RNAi, tumor microenvironment, KRAS, solid tumor

## Abstract

The distribution of drugs within solid tumors presents a long-standing barrier for efficient cancer therapies. Tumors are highly resistant to diffusion, and the lack of blood and lymphatic flows suppresses convection. Prolonged, continuous intratumoral drug delivery from a miniature drug source offers an alternative to both systemic delivery and intratumoral injection. Presented here is a model of drug distribution from such a source, in a multistep process. At delivery onset the drug mainly affects the closest surroundings. Such ‘priming’ enables drug penetration to successive cell layers. Tumor ‘void volume’ (volume not occupied by cells) increases, facilitating lymphatic perfusion. The drug is then transported by hydraulic convection downstream along interstitial fluid pressure (IFP) gradients, away from the tumor core. After a week tumor cell death occurs throughout the entire tumor and IFP gradients are flattened. Then, the drug is transported mainly by ‘mixing’, powered by physiological bulk body movements. Steady state is achieved and the drug covers the entire tumor over several months. Supporting measurements are provided from the LODER™ system, releasing siRNA against mutated KRAS over months in pancreatic cancer *in-vivo* models. LODER™ was also successfully employed in a recent Phase 1/2 clinical trial with pancreatic cancer patients.

## INTRODUCTION

Solid tumors appear in over 85% of human cancers. In systemic administration, therapeutic agents, namely anti-cancer drugs, whether unmodified, modified, non-encapsulated, or encapsulated, are exposed to processes in the bloodstream, including endocytosis and enzymatic degradation. More than 95% of the intravenously (IV) administered, encapsulated anti-cancer drugs are found to accumulate in other organs, in particular the liver, spleen, and lungs, which accounts for their toxicities [1]. A fraction of the released drug is also cleared, mainly in the kidneys. The overall effect is strong elimination and degradation prior to reaching the target tissue.

The remaining fraction can extravasate to the tumor via the enhanced permeability and retention (EPR) effect, as demonstrated in the long-circulating nanomedicines Doxil^®^ and Abraxane^®^; however, EPR efficiency in clinical settings is highly variable [1–3]. For the fraction remaining after extravasation, drug transport within tumors is further challenged by the specific tumor microenvironment conditions. Tumors dramatically differ from healthy tissues. Cancer cells and the extracellular matrix are abnormally dense and the tumors virtually are impervious to diffusion and convection. High levels of collagen and stabilized polysaccharide networks (hyaluronate and proteoglycans) within the collagenous fibers increase resistance to interstitial transport [4–6]. An inverse correlation between tumor packing density and drug penetration has been demonstrated [7]. The tumor microenvironment and/or tumor cell features, for example in models of breast cancer, can lead to variability in delivery and efficacy of nanoparticles (less prevalent for small-molecule drugs) and consequent variability in therapeutic outcomes [8]. Moreover, solid stress derived by rapidly proliferating cancer cells compresses blood and lymphatic vessels. As the cores of tumors are nearly avascular, the absence of functional lymphatic vessels results in interstitial hypertension [9]. Decades ago, the interstitial fluid pressure (IFP) in tumor cores was shown to be elevated by ~10–20 mmHg compared to healthy tissue, thereby diminishing inward convective transport from the tumor peripheral arteries towards the inner core [10]. In a more recent study, an even more dramatic elevation of IFP, ranging from 75 to 130 mmHg, was observed in autochthonous pancreatic adenocarcinoma [11], vastly exceeding typical arteriolar and capillary pressures of 40–80 mmHg and 15–40 mmHg, respectively. The authors concluded that pressures of this magnitude present not only major impediments to delivery and convection of small molecules, but also imply a profound reorganization and remodeling of the tumor architecture and the forces within it.

After more than three decades of research, the general view is that transport of small molecules (MW < 1 kD) within the tumor interstitial space occurs mainly by diffusion, whereas transport of larger molecules and nanoparticles would be extremely slow and ineffective as well as highly dependent on conditions affecting diffusion and convection [3, 12, 13]. Altogether, such abnormal physiological conditions in solid tumors present a strong barrier against effective systemic drug delivery strategies to treat solid tumors, even if the therapeutic agent crosses the bloodstream and extravasates into the tumor.

We propose here that a system for local and prolonged drug delivery that is placed within a solid tumor can solve such drug distribution challenges. Local (or focal) and prolonged delivery has been studied *in-vivo* and in humans [14–18]. Efficacy was shown to be dependent on design considerations, including the type of drug, materials, dimensions, drug load, release curves, and release period. For example, simulations of intratumoral drug distribution indicated that paclitaxel released from hydrogel (OncoGel^®^) and carmustine released from Gliadel^®^ wafers are characterized by similar therapeutic penetration depths (1–2 mm), but by varying durations of effective therapeutic concentrations (30 days vs. 4 days, respectively).

In this study, we present a model in which drug transport and distribution are described to occur in three consecutive steps named ‘Priming’, ‘Convection’ and ‘Diffusion + Mixing’. Unlike intratumoral injection, the drug is released “dry” (not associated with a fluidic form such as suspension or gel) to avoid fast clearance to the peripheral arterioles due to high IFP at the core. The drug that is released at an earlier stage, typically on the first day, modifies the immediate tumor microenvironment and paves the way for drug molecules that are released at later times to penetrate further. Such a pharmacodynamics role in continuous (non-injected, non-fluctuating) and prolonged drug delivery is essential, as it enables effective convection. It is demonstrated here that drug distribution by convection solves inefficiency of diffusion and would lead to cell death throughout the entire tumor. Indeed, it would be worth to include such a delivery mode, and the modifications in the microenvironment, in further studies based on detailed numerical simulations [19, 20].

As a supporting case study, we describe a system for prolonged delivery of short interfering RNA (siRNA) within murine pancreatic tumors via the LODER™ technology. The LODER™ (Local Delivery EluteR) is a millimeter-scale bio-polymeric drug delivery system that releases siRNA against G12D mutated KRAS(a drug called siG12D) over the course of four months [21]. The LODER™ dimensions and the surface area remain unchanged and constant over the entire release period. Unlike nanoparticles or micelles that migrate in the tissue, the drug is released from a fixed location in the tumor, where LODER™ was inserted. To facilitate the priming-convection-mixing steps, the release rate was shaped and fine-tuned by optimizing chemistry and manufacturing. In the example case presented here, approximately 20% of the drug load was released during the first day to support ‘priming’, another 30% was released during the first week to assure the process of increasing void volume and drug coverage of the whole tumor, and the rest was released as a zero order linear rate over the following four months. Later, LODER™ is dissolved in the tissue. It was demonstrated that the LODER™ surface remains clear, without significant accumulation of a strong stromal and/or protein blocking layer. Moreover, it was demonstrated that *in-vivo* LODER™ preserved the siRNA drug, either in modified or unmodified form, against enzymatic degradation for several months. For clinical use, 350 μg of siG12D-LODER™ was designed to be inserted by 19Gauge biopsy needles with an Endoscope Ultrasound (EUS) procedure and was optimized in terms of physical dimensions, ease of insertion, and regulatory considerations. The therapeutic effect of siG12D-LODER™ has been assessed by subcutaneous (ectopic) and orthotopic xenograft and synograft models [21], as well as in a phase 1/2a clinical trial with pancreatic cancer [22].

In clinical practice, in addition to local and prolonged drug delivery, we expect simultaneous systemic delivery of other drugs. It is presumed that the local delivery described here can also increase void volume and reduce local IFP in human tumors. If other drugs are delivered systemically, then the efficiency of drug penetration from the peripheral arterioles is expected to increase, hence facilitating drug permeation into the entire tumor mass. Such drugs include FOLFIRINOX [23], Gemcitabine/Abraxane^®^ [24] and immunotherapies including recent checkpoint inhibitors/immune modulators drugs in clinical trials, such as MEDI4736, a PD-L1-targeting antibody [25], Pembrolizumab (Keytruda^®^, MK-3475), an anti-PD-1 antibody [26], Ipilimumab (Yervoy^®^), an anti-CTLA-4 antibody [27], MPDL3280A, an anti-PD-L1 antibody [28], PF-05082566, an anti-4–1BB/CD137 antibody [29], Urelumab (BMS-663513), another anti-4–1BB/CD137 antibody [30], and more drugs in trials in the areas of novel adoptive T cell therapy and vaccines, monoclonal antibodies and cytokines.

## MODEL AND RESULTS

### Drug transport in tumors

#### Transport equation

In order to study the dynamics of drug delivery within tumors, we assessed the contribution of each factor in the transport equation, namely convection, diffusion, and reaction. Adopting a macroscopic approach, we will concentrate on a one-dimension linear (when needed, it will be converted to radial) case and select numerical values that characterize the typical tumor microenvironment. Nowadays, most of modeling and simulations of drug transport in solid tumors assume systemic delivery. In systemic delivery, drugs are transported by blood flows through capillaries located mainly in the tumor periphery, and are required to cross the capillary walls. The drugs then are needed to be transported inward to the tumor core, in general upstream against the IFP gradients. In our model, however, the drug source locates at the tumor core and the drug is continuously released directly into the tissue, and is distributed downstream along the IFP gradient, without the need to penetrate a capillary wall.

The change rate of the drug concentration *c* is given by the diffusion-convection-reaction equation, which, for a constant diffusion coefficient, can be read as

$$\frac{\partial \text{c}}{\partial \text{t}}=-\nabla *({\text{J}}_{\text{convection}}+{\text{J}}_{\text{diff}})+R$$

where J_convection_ = ($\vec{v}$*c) is the convection (or ‘advective’) flux, here $\vec{v}$ is a fluid velocity vector; J_diff_ = −D∇c is the diffusion flux, here D is the diffusion coeffecient; *R* = a general reaction term for drug sources and sinks.

Drug convection within a tumor depends on pressure differences, hydraulic conductivity, and external body movements that generate “mixing” or “stirring” flows in the interstitial fluid (osmotic pressure gradients are ignored here, as only flows within the interstitium are of interest, not those at the vascular-interstitium boundaries). As the IFP in a tumor core is higher than in the peripheral tissues, the net convection flow in the tumor interstitium is expected to be outwards from the core. Diffusion flux depends on concentration gradients and diffusivity. In our model, the “*R*” variable relates to the pharmacokinetics terms absorption and elimination in the interstitium, specifically to degradation in extracellular space and sinking into the local blood system. Here, we study the case where the effect of these processes is constant (but not negligible). We substitute *R* = constant and define:

Hydraulic conductivity coefficient: K = γK_0_, where $K_{0}\;\equiv \;10^{-7}\frac{cm^{2}}{mmHg*\sec};$

Hydrostatic pressure difference: ${\begin{array}{c} \nabla {\text{p}}_{\text{hyd}}=\delta \nabla p \end{array}}_{0},\text{ where }{\begin{array}{c} \;\nabla p \end{array}}_{0}\equiv 10\text{ mmHg}$

Diffusion coefficient: $\text{D}={\text{βD}}_{0\;},\text{ where}\equiv 10^{-6}\frac{{\text{cm}}^{2}}{\text{sec}};$

Neglecting mixing, convection is hydraulic and can be evaluated by Darcy's law, which was found to be sufficient in spherical symmetric steady-state flows of velocities smaller than ~1 mm/day of interstitial fluid [31]. Furthermore, we assume that the same length scale ∇1 is valid for both the pressure gradient and concentration gradient between tumor core and edge, and we approximate the hydraulic convection velocity by the Darcy equation:

$${\text{V}}_{\text{Convection},\text{ hyd}}=-\text{K}\nabla {\text{p}}_{\text{hyd}}\approx \text{K}\frac{{\text{p}}_{\text{core}}-{\text{p}}_{\text{edge}}}{\nabla \text{l}},$$

and the concentration gradient by $\nabla \text{c }\approx \frac{{\text{c}}_{\text{core}}-{\text{c}}_{\text{edge}}}{\nabla \text{l}}\approx \frac{{\text{c}}_{\text{core}}}{\nabla \text{l}}$.

With the assumptions above, the dimensionless Péclet Number *Pé* = (convection flux/diffusion flux = V*c/D∇c) becomes independent of concentration and length scale, and thereby presents a very helpful intuitive tool. *Pé* is given by

Péclet Number $Pé=\;\frac{\text{γδ}}{\text{β}}P_{é0};\text{ where }P_{é0}\equiv \frac{{\text{K}}_{0}\nabla {\text{p}}_{0}}{{\text{D}}_{0\;}}\;=1$

Notably, in the analysis presented here we use a linear gradient of IFP rather than a step function. Step functions (with rounded edges) are widely used in simulations of systemic delivery [20, 32]. In step functions the IFP profiles essentially are a plateau of constant value from the tumor center to the tumor edge (where a tumor tissue meets a vessel wall) and sharply drop along the vessel wall. Indeed, in such simulations the drug transport, which is oriented inward upstream against the IFP gradient, was found to be dominated by diffusion and not convection [32].

At the *Pé* ≫ 1 limit (γδ ≫ β), the transport is dominated by convection, and at the *Pé* ≪ 1 limit (γδ ≪ β), the transport is dominated by diffusion. In the tumor microenvironment, the convection *Pé* > 1 case is associated with lymphatic and blood flows. In the avascular core of the tumor, mainly the lymphatic drainage is expected to be effective, provided that the void volume (i.e., the intercellular space which does not contain cells, where transport of drugs can take place) is sufficiently large. Void volume can be deduced by the observational two-dimension “cell packing” parameter, defined as the product of cell density times the average area of a single cell. Two-dimensional cell packing can be measured on histopathological slides by using the following equation:

**Packing parameter** (2D) = [(*number of nuclei in area*) × (*average cell area*)] in the measured area

We obtained the relative void volume from the difference between the entire measured area and the packing area, powered by 3/2 for conversion to three dimensions, as follows:

**Void volume** = [1 − *Packing parameter(2D)/the measured area*]^3/2^

**Hydraulic conductivity**: The hydraulic conduc-tivity in tumor tissue depends on physiological conditions, including the concentration of collagen and hyaluronan [9, 33]. The hydraulic conductivity coefficient is expected to be in the range of K = 0.4–2.5 × 10^−7^ cm^2^/mmHg/sec (0.4 < γ < 2.5) [4, 19, 34].

**Diffusion coefficient**: The precise measurement of the diffusion coefficient of drugs, and specifically of siG12D in the tumor microenvironment, is beyond the scope of this paper. Still, the upper limit of the diffusion coefficient can be assessed by the Stokes-Einstein approximation:

$$D=\;\frac{kT}{6\pi \eta R_{H}}$$

where *k* is Boltzmann's constant; *T* is the temperature, η is the viscosity of water, and *R_H_* is the hydraulic radius, estimated by

$${\text{R}}_{\text{H}}={\left[\frac{3(\text{MW})}{4\pi \text{N}\rho }\right]}^{1/3}$$

where N is Avogadro's number, ρ is the specific density, and MW is the molecular weight.

### Model

**Figure 1:**
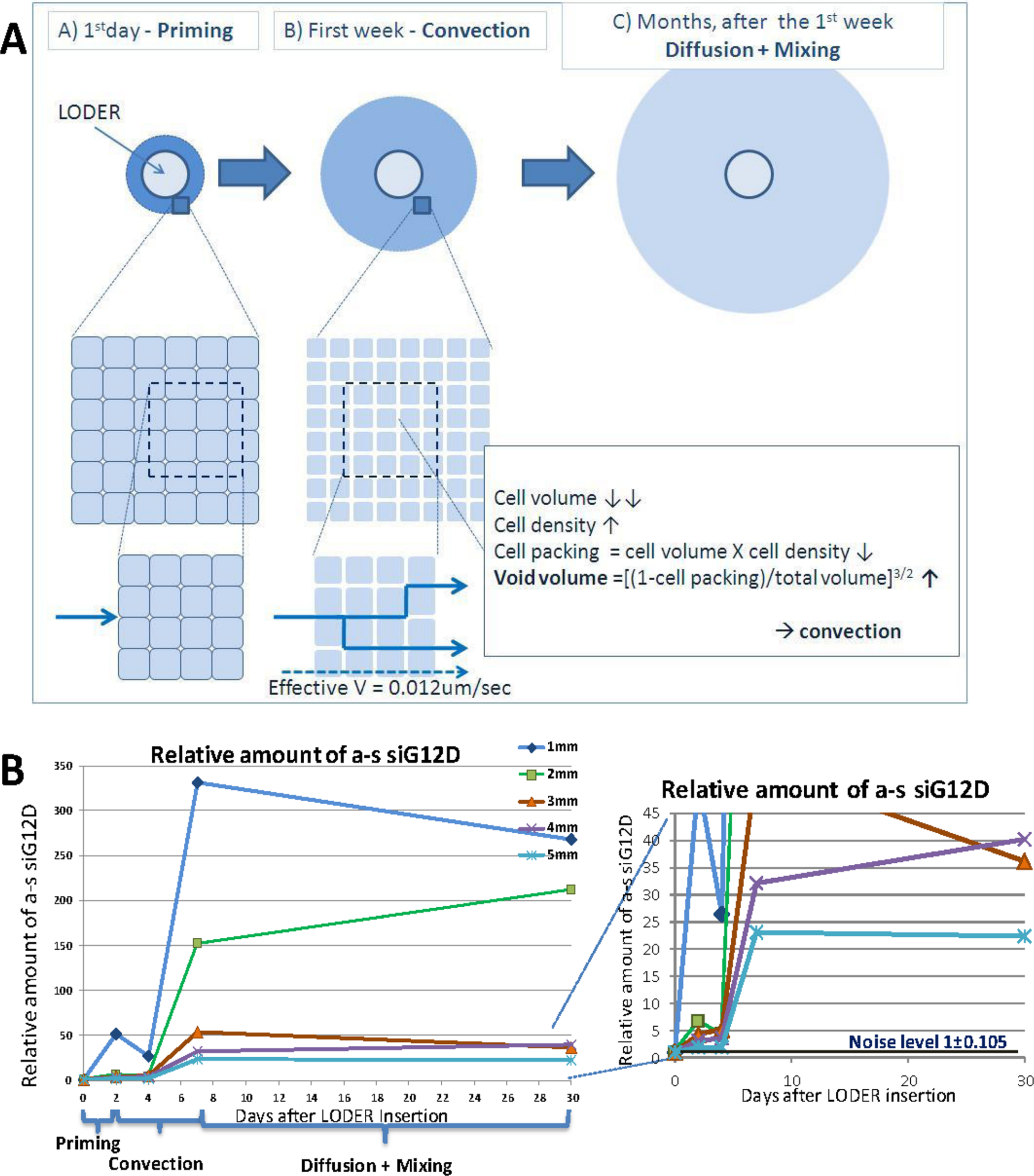
Drug transport is described as occurring in three consecutive steps: ‘priming’, ‘convection’ and ‘diffusion + mixing’ The drug that is released at an earlier stage, typically the first day, paves the way for the subsequent distribution of more drug molecules at later times by modifying the microenvironment physiological conditions **A–B**. **(A)** Model **(B)** The graphs show the average relative level of antisense (a-s) siG12D detected at days 0, 2, 4, 7 and 30, at concentric rings of 1 mm width and radius of 1, 2, 3, 4 and 5 mm from the LODER™. Each point shows an average of at least two independent measurements. Methods: subcutaneous (SC) or orthotopic Panc-02 tumors were treated with siG12D-LODER™. As the presented model is general for solid tumors, we use here data obtained in SC and orthotopic models. Two, four and seven (in SC model) or 30 (in orthotopic model) days after the insertion, mice were sacrificed, tumors were formalin fixed and paraffin embedded (FFPE), and tissue sections of 5 μm were prepared. RNA was extracted at a certain distance from LODER™. Relative amounts of siG12D antisense strand were measured using Real-Time PCR. RNU6 microRNA was used as an endogenous control. siG12D levels were calculated relative to the detected level in untreated or placebo-LODER™-treated tissues.

**Figure 2:**
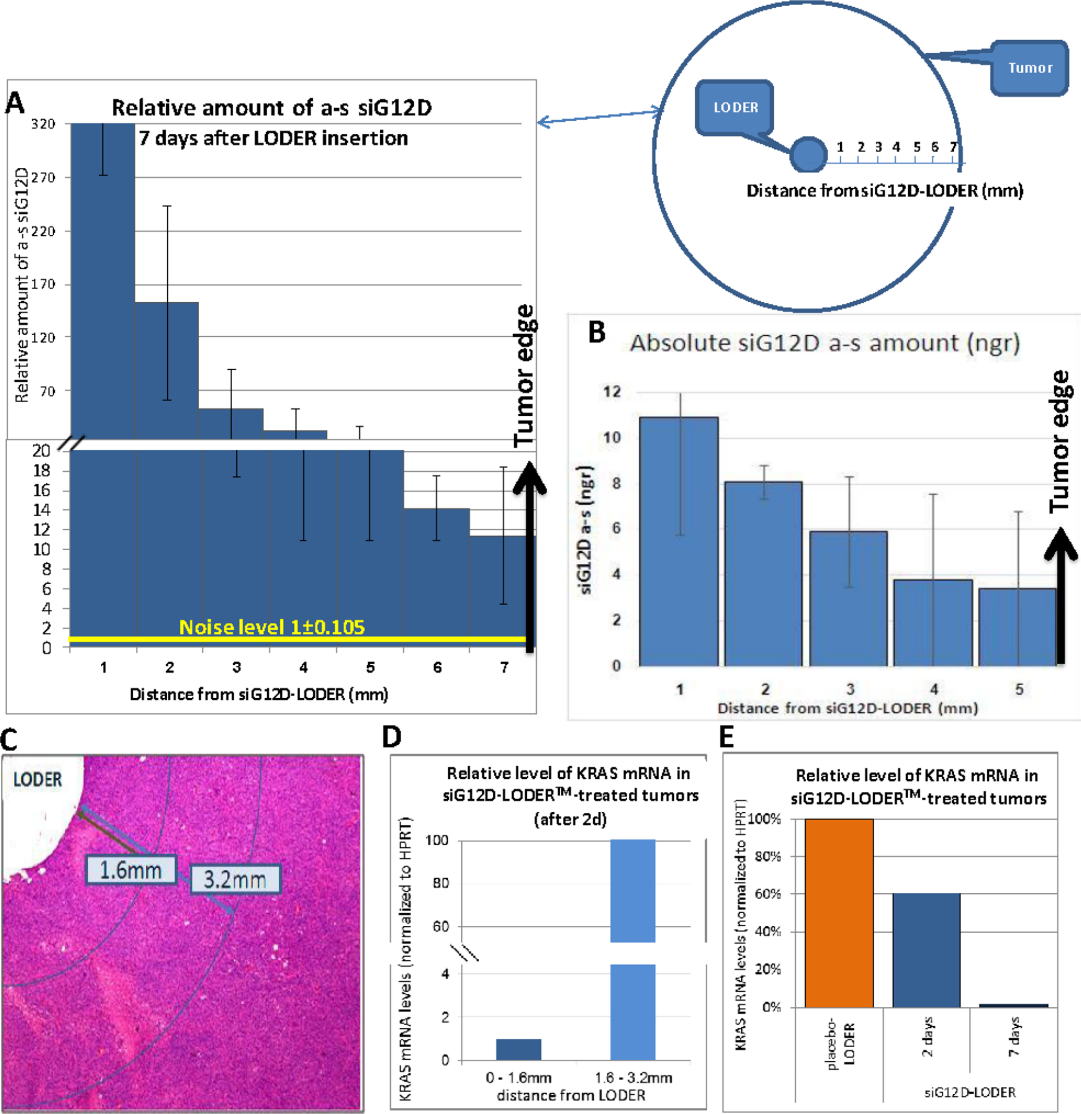
SC or orthotopic Panc-02 tumors were implanted with siG12D-LODER™ Two, four, seven and 30 days after the insertion, mice were sacrificed, tumors were subjected to FFPE, and tissue sections of 5 μm were prepared. RNA was extracted at certain distances from LODER™. Relative amounts of siG12D antisense (a.s.) strand was measured using Real-Time PCR. RNU6 microRNA was used as endogenous control. siG12D levels were calculated relative to the detected levels in untreated or placebo-LODER™-treated tissues. **A**. Average relative detected levels of antisense siG12D ± SEM at certain distances from the LODER™ at day 7 (presented here is a representative case – the values of two, four, seven and 30 days are presented in Figure 1B). **B**. Absolute levels of antisense siG12D ± SEM detected at day 7 at 1, 2, 3, 4 and 5 mm distances from the siG12D-LODER™. **C–E**. Relative quantitation of KRAS mRNA (normalized to HPRT) in siG12G-LODER™-treated tumor tissues. **(C)** Representative histological slide showing measurement areas. **(D)** Relative amount of KRAS mRNA as a function of distance from siG12D-LODER™. Shown here are representative results of treated tissue. **(E)** Relative KRAS mRNA levels as detected at two and seven days after LODER™ insertion. The results were normalized to the level in placebo-LODER™-treated tissue seven days after the insertion. Shown here are representative results of treated tissues.

**Figure 3:**
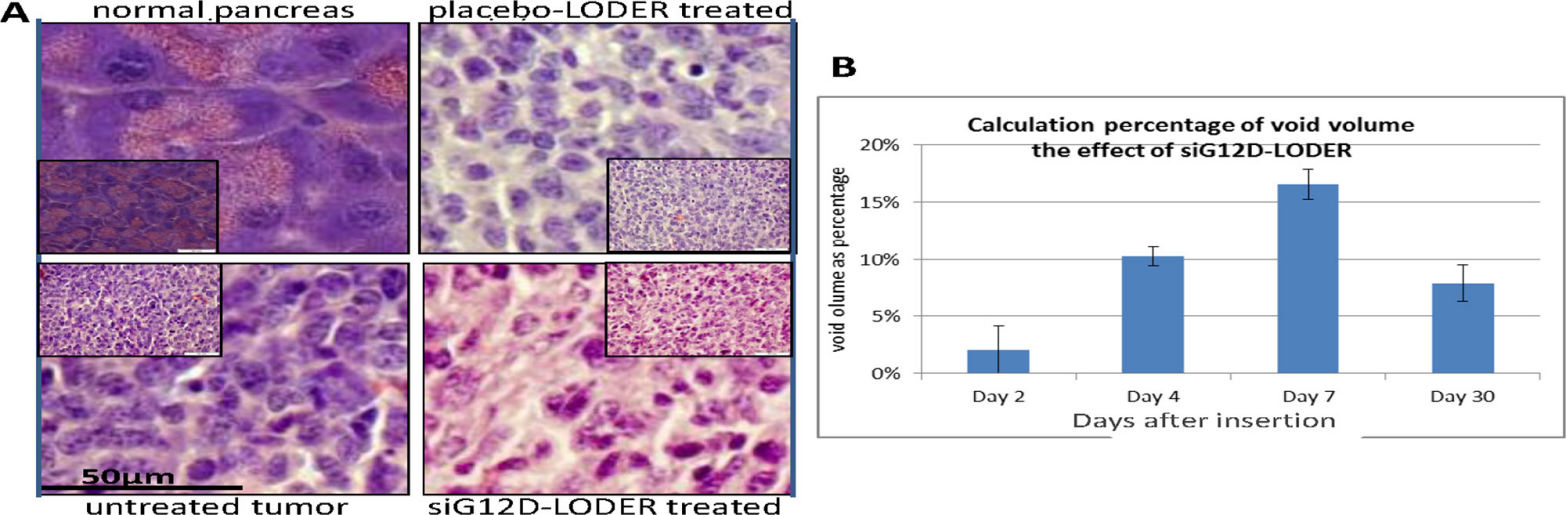
Void Volume **A**. Mice were orthotopically implanted with Panc-02 tumor allografts that were left untreated (u/t), treated with an placebo-LODER™ or treated with siG12D-LODER™. One month after the insertion, mice were sacrificed and tumor tissue was subjected to FFPE and H&E. Immunohistochemical staining was carried outon 5 μm tissue sections. The slides were then imaged microscopically. **B**. Panc-02 SC tumors were treated with siG12D-LODERs. On days 2, 4, and 30, mice were sacrificed and tumor tissues were subjected to FFPE and H&E. Immunohistochemical staining was performed on 5 μm tissue sections. The slides were then imaged microscopically. The graph represents average cell area ± SEM. Cell density was calculated as number of nuclei/(10 μm)^2^. All the measurements were performed on non-necrotic tumor tissue sections. The graph represents average percentage of void volume in tested tissue ± SEM. Void volume formula is: (1 − [(number of nuclei/(10 μm)^2^)*average cell area)/calculated area])^3/2^.

**Figure 4:**
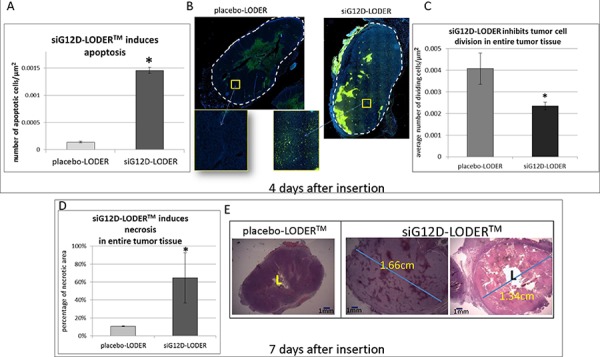
Effects of apoptosis (TUNEL) and necrosis (H&E) Panc-02 cells were injected SC into the right flanks of C57/Bl6 mice (syngeneic SC model). When tumors reached ~1 cm^3^, mice were divided into groups so that the average tumor volume in all groups was the same. Indicated LODERs were inserted intratumorally, one LODER™/tumor. Four or seven days after the insertion, mice were sacrificed, tumors were subjected to FFPE, and 5 μm tissue sections were prepared. **A–B**. Detection of apoptotic nuclei. Tumor tissues were immunostained four days after insertion using the TUNEL method, which detects apoptotic nuclei. Slides were visualized using a Nikon microscope, and the number of apoptotic nuclei was counted using the N is elements computer program (Nikon Instruments Inc.). (A) Average number of positive (apoptotic) nuclei per μm^2^ ± SEM; (B) Representative tissue sections, TUNEL IH staining; **C**. Four days after insertion, dividing cells in tumor tissue were detected by CDC47 immunostaining. The graph represents the average number of dividing cells/μm^2^; **D–E**. To detect necrotic regions, tumor tissue slides were H&E stained seven days after insertion. The slides were visualized using a Nikon microscope. Necrotic area was calculated using the N is elements computer program (Nikon Instruments Inc.) (D) The graph shows the percentage of necrotic area ± SEM; (E) Representative tissue section. *p < 0.05 based on Student's *t*-test.

We present a model of drug transport in solid tumors from a drug delivery system that is inserted intratumorally and releases drug continuously during a period of months. The model is illustrated in Figure 1A and is justified to a large extent by measurements of relative and absolute drug concentration as a function of time and distance, presented in Figure 1B and Figure 2A–2B, together with supporting measurements of changes in microenvironment (Figure 3) and effect of drug (Figure 4). The drug transport is described as occurring in three consecutive steps - ‘Priming’, ‘Convection’ and ‘Diffusion and Mixing’.

**Step 1 – Priming** At the onset of drug release from the drug delivery system, in a highly dense tumor core, the effect is very local. Drug transport is limited to the cell layers closest to the system. Drug molecules will in part penetrate into such cells and lead to apoptosis and necrosis.

**Step 2 – Convection** (Péclet Number *Pé* > 1) void volume increases, drug is transported outwards, associated with lymphatic fluid that streams from the inner core to the entire tumor downstream along the tumor's hydrostatic pressure gradient.

**Step 3 – Diffusion and Mixing** - cell death by apoptosis (in addition to necrosis) occurs throughout the entire tumor, gradients of pressure are flattened, and hydraulic convection becomes ineffective. Microenvironmental conditions favoring diffusion become more effective than in an untreated tumor and likely also compared to diffusion in healthy tissues. In addition to diffusion, it is proposed here that bulk body movements now are facilitate drug distribution by generating ‘mixing’ flows. As long as the drug delivery system releases the drug at a stable rate, the drug transport is in steady state, and levels of drug concentration and spatial concentration gradients throughout the tumor remain constant.

### Drug distribution from siG12D-LODER™

The example case study presented here is of prolonged delivery of siRNA from the siG12D-LODER™ delivery system in mouse pancreatic tumor models. Unlike systemic delivery, LODER™ releases the drug within the tumor core, and the drug distribution is free from the main obstacle of crossing blood vessel walls. Moreover, unlike local intratumoral injection, the drug when is released here is not associated with a fluidic form such as suspension or gel, and is therefore free from fast drainage to lymphatic or blood vessels. Unlike both cases, the drug is released continuously over months. Such a long period of release is designed to be orders of magnitude greater than other crucial time scales, including cell division (~1–2 day) [35], induction of apoptosis by siG12D (<3–4 day) [21], and pressure dissipation (typically a week, per this paper).

#### Pharmacokinetics and pharmacodynamics

Estimation of pharmacokinetics (PK) and pharmacodynamics (PD) values, including the “*R*”reaction term, cell uptake, and number of molecules bound in RISC per cell, can be performed by comparing the measured absolute amount of drug to the total amount of drug released from LODER™ at the relevant period of time (RISC stands for RNA-Induced Silencing Complex, the complex where RNA interference (RNAi) occurs). It was demonstrated previously [21] that uptake of naked siRNA can occur in solid tumors, specifically in*in-vivo* models of pancreatic cancer; however, the uptake efficiency was not assessed by the authors. Cell uptake of naked RNA and DNA can be non-negligible in the abnormal conditions of the tumor microenvironment. Some mechanisms, not explored here, can increase membrane permeability, including high pressure [36, 37], low pH levels [38], *in-situ* complexation with ammonia (Silenseed, in preparation) due to the very high level of ammonia in tumors [39], or transient phenomena associated with cell division. Notably, it has also been claimed that lipophilicity and backbone flexibility can enhance tissue penetration of RNAi compounds with small duplex regions of < 15 base pairs [40].

The relative amount of siG12D drug transported from a LODER™ to the surrounding tumor tissues was measured as a function of radial distance and time (Figure 2A). Subcutaneous (SC) or pancreatic orthotopic tumors were induced in syngeneic mice. To obtain histological features that optimally can present advanced pancreatic cancer we mostly have selected the Panc-02 cell line. Panc-02 is known to be invasive and metastatic with a fast evolving vasculature network [41]. Notably also in the Panc-1 cell line, it was demonstrated that orthotopic *in-vivo* tumors mimic histological features of advanced pancreatic cancer. Moreover, both orthotopic and ectopic (SC) Panc-1 tumors showed chaotic and disorganized blood vessels [42]. Mice were divided into three similar treatment groups: ‘untreated’, ‘placebo’ (treated with LODER™ without drug), and ‘treated’ (treated with siG12D-LODERs). In LODER™-treated groups, a single LODER™ was intratumorally inserted with placebo-LODER™ or siG12D-LODER™ containing 5 μg of siG12D. Mice were sacrificed at four and seven days post-insertion in the SC model and 30 days post insertion in the orthotopic model. Tumor tissue sections were scraped radially in concentric rings of 1 mm width, at 2, 3, 4 and 5 mm radial distances from the LODER™ emplacement. A siRNA-enriched fraction was purified for each ring.

Measurements have been performed using relative quantitative Real-Time PCR by detection of the antisense(a-s) strand of the siG12D molecule and presented as a function of time and distance from the LODER™ (Figure 2A). The results were normalized to the level of RNU6 microRNA [43]. The reaction in use is sensitive only to the siG12D antisense and insensitive to both the double-stranded intact siG12D molecule and its degradation products. We assume that the detected a-s siG12D molecules exclusively represent the molecules bound to active RISC.

The absolute amount of drug as a function of distance from LODER™ was deduced by calibrated absolute Real-Time PCR. Data from these experiments are shown in Figure 2B. For assessment of drug transport, the measurements of absolute drug levels can be considered as a lower limit, as most of siG12D molecules delivered from LODER™ are expected to degrade intercellularly in the interstitial space, and additional amounts of the molecules that penetrated into cells will degrade intracellularly prior to being uploaded to the RISC where RNAi occurs. Only the antisense of the double-stranded siRNA that is trapped and bound within the RISC complex remains stable for many hours. Therefore, using calibrated RT-PCR measurements we expected to measure, in essence, only those antisense molecules. The calibration error was found to be 13.2%.

Based on the considerations above we studied the ranges of the reaction parameter (hereafter “REACTION”) and the amount of drug uptake into cells (hereafter “UPTAKE”). As shown in Figure 1B, after a week, drug concentration and concentration gradients become constant. We therefore selected the data of day 7 (Figure 2A) and performed PK/PD assessments. The REACTION parameter presents the accumulated drug lost from releasing at the LODER™ surface until being up-taken into cells, including drug sinking into the blood and drug degradation in the intercellular space. It is given by:

REACTION=TOTAL−UPTAKE

where hereafter “TOTAL” stands for the total drug released from LODER™ into the tumor in the first 7 days. In this exemplary *in-vivo* study we used LODER™s containing 5 μg that release 26.7% ± 7.1% [22] in the first 7 days. We obtained TOTAL = 1.335 ± 0.095 μg.

The cell uptake term is given by

$$\text{UPTAKE}={\text{R}}_{\mathrm{ic}}+2*\text{BOUND}$$

where R_ic_ stands for all intracellular reaction processes occurring within the cell excluding binding to RISC. Such processes include binding to receptors and RNA degradation. Hereafter “BOUND” stands for the amount of drug bound to RISC in cells in the entire tumor (to compensate, we multiplied BOUND by factor 2 as only the antisense of the double strand siRNA is bound into RISC). Direct measurements of R_ic_ are beyond the scope of this paper. So far, the intracellular reaction of siRNA is not well established and was assessed per the specific delivery vehicle and specific RNAi molecule design [44]. We therefore substitute five “test-plug” values for intracellular degradation efficiency (hereafter “X%”): R_ic_ = X%*UPTAKE; where X% = 1%; 25%; 50% 75%; and 99%, and received:

UPTAKE=2*BOUND/(100%−X%)

We obtained the BOUND value by measuring the total absolute amount of drug in the central slide of the tumor, and extrapolating from slide volume to a sphere volume of the same radius. The total amount of drug as measured at day 7 and integrated over five concentric rings of radius ranging from 1 mm to 5 mm (5 mm radius represents the tumor edge in this *in-vivo* case) was 2.010 × 10^−5^ μg ± 13.2%. Extrapolating to a sphere, we obtained BOUND = 1.398 × 10^−3^ μg ± 13.2% (we assume the PCR calibration error is dominant compared to other errors in this part).

Finally, we converted the parameters described above to relative terms R ≡ REACTION/TOTAL and relative uptake (= UPTAKE/TOTAL) (Table 2).

Under the assumptions presented here we obtained ranges of the relative reaction term: 79.05% < *R*< 99.79%, and of relative uptake 0.21% < UPTAKE/TOTAL < 20.95%. The estimated relative error is 15% of such values, obtained from the combination of the two independent errors of measuring TOTAL (+/− 7.1%) and BOUND (+/− 13.2%).

Not surprisingly, we found that the reaction *R* was high, *R* > 79%, as is expected from fast degradation of RNAi, and that relative uptake of naked (not conjugated, not encapsulated) siRNA is low, below 21%. Still, because of the high number of molecules released per cell, the process was found to be efficient in achieving RNAi-based effects throughout the entire tumor. Specifically, we deduced the number of cells in the tumor from the [Tumor volume/Cell volume] ratio, where the cell volume is calculated by [measured average cell surface]^3/2^ (Figure 3A–3B). With the obtained total number of cells and the considerations above we found that the average number of molecules bound to RISC per cell is 284.7 ± 37.6, which is in good agreement with the measured range of 20–5000 [45] required for effective RNAi.

#### Void volume

Void volume can be used to assess the penetrability of drug into tumor tissues. Of note, void volume should not be used to assess global tumor response, as measurements are performed only in non-necrotic tumor tissue sections, which are highly populated with cancer cells. We've found that measurements in highly necrotic tissues, either resulted from drug effect or not, are irrelevant. To calculate void volume, and to double check, we used both syngeneic pancreatic orthotopic and SC models. Tumor allografts were treated with siG12D-LODERs, placebo-LODERs without any agent, or left untreated, or treated with a control siLuc-LODER^TM^, which contains siRNA targeting the luciferase gene. In the first set of studies (siG12D-LODER^TM^, placebo and untreated) mice were sacrificed seven and 30 days post-insertion in SC and orthotopic models, respectively. Paraffin-embedded tumor tissue sections were prepared, and H&E immunohistochemical staining was performed. Slides were imaged microscopically in order to deduce the cell surface density and average cell area. The relative void volume = (Void volume)/(Tumor volume), was calculated by: relative void volume = [1 − (cell surface density * average cell area)/tumor area])^3/2^. Relative void volume was found to increase during the first week from a fully resistant tissue, as measured in untreated tumors, of packing parameters ~0%, to ~10% on day 4 and ~17% in day 7, and it remained rather stable, with a slight decrease to 7.9% ± 1.6% after 30 days (Figure 3B).

In an additional study we tested the effect of siG12D-LODER^TM^ vs. siLuc-LODER™. LODERs were intratumorally inserted into subcutaneous tumors originated from Capan1 cells constitutively expressing luciferase gene. Mice were sacrificed twenty days post treatment, and tumors histology tissue slides were analyzed. The results reveal that siG12D-LODER^TM^ generates an increase of 17.75% +/− 4.34% in void volume, while siLuc-LODER™ presents none or a very small increase of 2.98% +/ − 2.96% in void volume, perhaps an off-target and/or immune result of the non-specific siLuc.

Cell apoptosis and necrosis can explain the increase in void volume. We've noted that after one week levels of mRNA siG12D are significantly reduced as compared to the levels after two days (Figure 2E). We estimated the amount of cell death by measuring apoptotic and necrotic tissue fractions. Here, SC syngeneic models were used. Tumors were intratumorally inserted with siG12D-LODER™ or placebo-LODER™. Immunohistological staining results of tumor tissues collected one week after the LODER™ insertion, revealed that in siG12D-LODER™-treated tumors significant induction of apoptosis (Figure 4A–4C) and necrosis (Figure 4D–4E) were detected [21].

#### Stages of drug distribution

**Priming** (~first 1–2days): The period of the priming stage is dictated by the time required for RISC loading and cleavage, estimated to be ~2 hours [46], and the time for apoptosis to occur, ranging from a few hours after RISC loading to a few days [21]. To accelerate priming, LODER™ was designed to release a burst of 20% of its drug load on the first day. For a LODER™ containing a total drug load of 5 μg (as was used in *in-vivo* studies) 20% drug burst would translate to 4 × 10^13^ siRNA molecules. Substituting a typical volume value of 1 mm^3^ for the closest layer into which the drug penetrates on the first day, which would contain 7.1 × 10^5^ cells (as obtained from the [cell density]^3/2^) in ‘untreated’ tumor, in Table 1), an average of 5.6 × 10^7^ siG12D molecules per cell is expected. For RNA interference to occur, the siRNA drug amount within the cytosol required for efficient RNA interference, as assessed by direct microinjection into the cell, was found to range from ~20 to ~5 × 10^3^ molecules per cell [45]. We see that such a range is smaller by 4–6 orders of magnitudes than the amount of drug molecules released per cell. Therefore, even if the cell relative uptake is much smaller than the lowest value in Table 2, 0.21%, the number of released molecules during that step will be sufficient to support immediate drug entrance into the cell layer at a depth of > 165 μm, allowing the priming step.

**Table 1:** Example of derivation of tumor void volume (on day 30)

	Cell Area (μm^2^)		cell density (nuclei/10 μm^2^)		Void volume (%)	
	Value	Error (+/–)	Value	Error (+/–)	1-Packing Parameter	void volume
**Normal pancreas cells**	**498.24**	30.22	**0.200**	0.011	0.49%	**0.03%**
**u/t tumor cells**	**123.39**	7.99	**0.828**	0.022	− 2.19%	**~0**
**Empty-LODER^TM^-treated tumor cells**	**150.46**	8.33	**0.723**	0.068	− 8.79%	**~0**
**siG12D-LODER^TM^-treated tumor cells**	**123.47**	5.5	**0.661**	0.029	18.33%	**7.9% +/** − **1.6%**

Mice were implanted orthotopically with Panc-02 tumor allografts and treated with placebo-LODERs without any agent, siG12D-LODERs, or left untreated (u/t). Mice were sacrificed one month post-insertion and tumor tissues were subjected to FFPE and H&E. Immunohistochemical staining was performed on 5 μm tissue sections. The slides were then imaged microscopically. Cell area was measured using the N is elements computer program.

**Table 2:** Reaction and uptake relative terms, as a function of relative intracellular degradation efficiency

X ≡ R_ic_/UPTAKE	*R* ≡ REACTION/TOTAL	relative uptake = UPTAKE/TOTAL
1%	**99.79%**	**0.21%**
25%	99.72%	0.28%
50%	99.58%	0.42%
75%	99.16%	0.84%
99%	**79.05%**	**20.95%**

Evidence of relative level of drug taken two days post LODER™ insertion seem to support such considerations (Figure 2B). Moreover, to assess the RNAi effect of siG12D drug we measured the relative amount of KRAS mRNA as a function of distance from siG12D-LODER™. Representative results of mRNA siG12D in an inner ring of radial distance 0–1.6 mm from LODER surface, compared with to the successive ring 1.6 mm–3.2 two days indeed show ratio of ~1:100 (Figure 2C–2D).

**Convection** (~first week): Our observations show that after one or two days post-insertion the drug expansion becomes linear in time, with an effective velocity of approximately 1 mm/day. We have speculated that the dominant factor is convection (Pé > 1), and not diffusion that typically has a square root dependence in time, or generally *t*^−n^ behavior where n ≥ ½. Cell death at the closest volume surrounding the LODER™ leads to increase in void volume by ~8%–17% (Figure 3), which would then enable fluid flow within the entire tumor, and drug convection.

To explore the conditions for convection-dominated transport (*Pé* > 1), we estimated the hydraulic velocity by the Darcy equation $\text{V}=\text{K}\frac{\text{Δp}}{\text{Δx}}$, using typical values of gradient of 12 mmHg over 1 cm, (δ = 1.2) and of hydraulic conductivity K = 1.0 × 10^−7^ cm^2^/mmHg/sec (γ = 1). From measurements in days 0, 2, 4, and in day 7 (depicted Figure 2A), we found an approximately linear propagation of drug with an observed velocity of ~1 mm/day (= 1.2 × 10^−6^ cm/sec). Multiplying by the 3D tortuosity factor [47], ranging from 2^1/2^ to 3, we obtained a lower limit for the range of drug velocity, yielding *V* > 1.7–3.6 × 10^− 6^ cm/sec.

As long as (δ > 1) it would be difficult to explain velocity by diffusion (*Pé* < 1; (γδ < β)) because the required values β > [1.2*(1.7–3.6)] are above the range expected for the diffusion coefficients for medium and large molecules with MW > 1kD. Specifically, one can substitute the molecular weight of siG12D MW = 13.5kD into the diffusion coefficient formula, yielding *D*_(siG12D)_ (in aqueous) = 1.19 × 10^−6^ cm^2^/sec. This approximation is in good agreement with values obtained for 20bp double-strand DNA measured by capillary electrophoresis [31]. Detailed diffusion measurements in tumor tissues [48] and extrapolation by the experimental power law *n*^−0.67^, where n is the number of base pairs, show lower values of D_(siG12D)_ to the range ~(0.17–1.1) × 10^−6^ cm^2^/sec, yielding the *a* value range of 0.17 < β < 1.1 (notably, higher values of interstitial velocities associated with capillaries were observed, probably related to strong osmotic pressure or response of the IFP to blood pressure changes [10, 12])

We therefore expect that the drug is transported mainly by convection at that stage. Hydraulic convection, as derived by gradients of IFP, can be efficient as long as the gradients Δp/Δx are higher than ~10–12 mmHg/cm.

**Diffusion and Mixing** (~weeks – months): Below IFP value of ~10 mmHg hydraulic convection seems to become less efficient. Notably, recent simulations [19] show that *Pé* ~ 0.1 indeed is a typical value in the tumor microenvironment. Still, drug diffusion alone might not be sufficient to explain the observed drug coverage of an entire tumor of radius *R* > 1 cm. The diffusion time scale for drug to cover the tumor is about a day or longer, while drug degradation might be faster. Our conclusion is that an additional mechanism, presumably mixing (or ‘stirring’), occurs at later stages.

The importance of mixing has been explored in several physiological conditions including the vitreous chamber [49]. In tumors, mixing is expected to be effective when random and temporal fluid flows can occur in the microenvironment. As shown in Figure 4, after the first week post insertion cell apoptosis and necrosis occurred over the entire tumor area. We presume that, thanks to the continuous drug release, such a wide and permanent cell death and a stable void volume can enable random flows.

**Figure 5:**
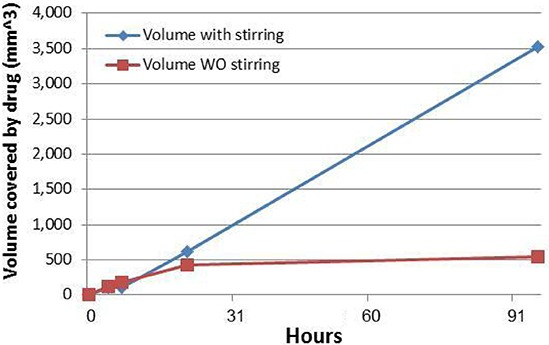
Comparison of drug distribution by diffusion vs. diffusion with mixing An amount of 5 μg of siG12D was added at the center of a plate with 3% Acrylamide gel in a two-dimension diffusion experiment. Diffusion alone was compared to diffusion+ mixing using 90RPM horizontal stirring. The siG12D molecules were visualized at specific time points using Ethidium Bromide by the ChemDoc MP Imaging System (BioRad). The area covered by drug was measured using the Image Lab program (BioRad) at time points 0, 4, 7, 21, and 96 hr. Volume was deduced by (= 4/(3*sqrt(π))*(measured area)^3/2^) in mm^3^. The Diffusion coefficient was found to be D_(siG12D)_ = 0.242 ± 0.214 × 10^−6^ cm^2^/sec.

To evaluate the importance of drug distribution for the siG12D drug by diffusion and mixing, as compared to diffusion only, we performed an *in-vitro* test of diffusion with and without mixing. We used 90RPM horizontal stirring of the siG12D drug in a two-dimensional model diffusion experiment in a 3% Acrylamide gel. The area covered by drug was measured at time points 0, 4, 7, 21, and 96 hr. Figure 5 depicts the average volume(= 4/(3*sqrt(π))*(measured area)^3/2^) in mm^3^. As shown, after the first day, diffusion is practically halted so that a volume of 1 cm^3^ will never be covered. By using the diffusion approximation for average displacement < r^2^ > = 2dD*t, where *d* is the dimension and t is time, we set *D* = (*measured area*/π)/(2*dt*), *d* = 2, yielding D_(siG12D)_ = 0.242 ± 0.214 × 10^−6^ cm^2^/sec; the measured parameter β = 0.242 is an interim value in the theoretical range 0.17 < β < 1.1 described above. With stirring, the drug is mixed in the gel and covers a volume of 1 cm^3^ in 1.3 days. Applying these results and the comparison of drug distribution by diffusion vs. diffusion with mixing (Figure 5) we conclude that mixing can be very efficient.

## DISCUSSION

Drug distribution within solid tumors is widely known to be inefficient, specifically when is based only on diffusion. Convection is also inefficient in systemic delivery, as the elevated IFP presents a barrier to transcapillary transport [50] and to inward flows upstream against the IFP gradients. Prolonged, continuous intratumoral drug delivery from a miniature drug source can lead to cell death and change in the microenvironment, which in a few days would increase the ‘void volume’ over the entire tumor from a neglected value to typically ~10%. We have developed a model of such prolonged and continuous intratumoral drug distribution, specifically a multiple-step process of drug delivery from a miniature drug-loaded system inserted in the tumor core. At the delivery onset immediately after insertion, a short stage of tissue ‘priming’, expected to result from tumor cell death, occurs at the cell layers closest to the delivery system, thus enabling the penetration of drug further into remote tumor tissue. Cell death at extended distances enlarges the intercellular void volume. After such a ‘priming’, transport of the drug during the coming days is mainly powered by hydraulic convection, aligned outwards from the core, downstream along the pressure gradients of the interstitial fluid. Later on, once cell death continuously occurs throughout the entire tumor and IFP can dissipate, pressure gradients are expected to be flattened and hydraulic convection would lose efficiency. Compared with untreated dense tumor tissue, diffusion may now be more efficient, following the microenvironment modifications by apoptosis and necrosis in the whole tumor volume. Still, drug diffusion alone might not be sufficient for a wide distribution, as the diffusion time scale to cover the tumor can be longer than a few hours or a day, while the drug degradation rate is expected to be faster. At the final stage, starting a few days post insertion and continuing over the course of months, we propose that a combination of drug ‘mixing’ associated with physiological bulk movements of the body and diffusion constitute the major mechanisms of continuous transport throughout the entire tumor.

Our measurements in a set of *in-vivo* studies support such a drug distribution model, including the importance of convection upon diffusion, and the time scale to achieve complete drug coverage in tumors. Of note, the ~13.5kD molecular weight of siRNA is an intermediate molecular weight between small molecules and large proteins and/or nanoparticles. As the diffusion coefficient is only weakly dependent on molecular weight (D × MW^−1/3^), the model presented here is expected to be applicable for a wide range of therapeutics.

It is also of interest to consider the increase in void volume and the flattening of IFP gradients as drivers for intratumoral migration of cells. Specifically, lack of infiltrating CD8+ effector T cells enable tumors to evade antitumor immune responses and to grow progressively [51–53]. In such tumors there is poor chemokine expression and also minimal presence of defined immune inhibitory pathways. It was speculated that these tumors also have denser stroma and alternative myeloid or macrophage populations. Here we speculate that extending the void volume of tumors, for example by methods described in this paper, can potentially accelerate infiltration of CD8+ effector T-cells and increase the effectiveness of anti-tumor immune responses. The concentric ring methods developed here to measure anti-tumor effects as a function of time and distance from LODER™ could be exploited in future studies, for example, in studying the temporal and spatial CD8+/Treg ratio in tumors.

An additional aspect to be further studied is the correlation between prolonged intratumoral delivery and lymphatic and interstitial flow in the tumor microenvironment and the effect on tumor immunology [54, 55]. In addition, it is expected that when additional chemotherapy or immunotherapy drugs are delivered systematically and simultaneously with the intra tumor treatment, the focal reduction of pressure gradients and increase in void volume could significantly improve the penetration of such drugs into tumor tissues. In summary, prolonged, continuous intratumoral drug delivery from a miniature drug source may present an alternative to current therapies for an effective treatment of solid tumors.

## SUPPLEMENTARY DATA


