# Supplementary material for: Multistep, effective drug distribution within solid tumors
Source: Oncotarget. 2015 Sep 22;6(37):39564–77. doi: 10.18632/oncotarget.5051 (PMC4741846; doi:10.18632/oncotarget.5051)
Supplement: Supplementary file 1 [file oncotarget-06-39564-s001.pdf]

SUPPLEMENTARY DATA

Loder™

LODER™ is a bio-polymeric cylindrical drug delivery system containing the siRNA drug that is released throughout a period of months into a tumor. Dimensions are optimized for insertion into the pancreatic tumor by a 19G endoscopic ultrasound needle (D~0.9 mm). The total drug loaded per LODER™ ranged from 0.5–15 µg for preclinical studies and was 375 µg for clinical use.

Cell lines and cell culture conditions

The Panc-02 cell line, a gift from Dr. M. Elkin, was cultured in RPMI-1640 medium, supplemented with 10% (v/v) heat-inactivated fetal calf serum (FCS), 2 mM glutamine, 100 units/mL penicillin and 100 µg/mL streptomycin. Cell culture was maintained in a humidified atmosphere of 5% CO_2 at 37°C.

Real-time PCR

RNA was isolated using TRIzol (Invitrogen, cat#15596) according to the manufacturer's instructions. cDNA was synthesized using the qScript™ microRNA cDNA synthesis kit (Quanta, cat# 95107). Real Time PCR was performed using the PerfeCTa SYBER Green SuperMix (QuantaBiosciences, cat# 95055-500). siG12D antisense was detected using the 5′-GTTGGAGCTGATGGCGTAG-3′ primer. RNU6 was used as an endogenous control and amplified using a commercial primer, PerfeCta micro RNA Assay RNU6 (Quanta Biosciences, RNU6). For absolute RT-PCR, known amounts of antisense molecule (5′-CUACGCCAUCAGCUCCAAC-3′ dTdT) were employed. To detect the relative level of KRASG12D mRNA, the following primers were used: Fw: 5′-C TTGTGGTGGT TGGAGCTGA-3′; Rev: 5-ATT ACT ACTTGTTTCCTG TAG G-3′. HPRT was used as an endogenous control: Fw: 5′-GCGATGATGAACCAGGTTATGA-3′; Rev: 5′-ATC-TCGAGCAAGTCTTTCAGTCCT-3′.

Animals

Female C57B/6 5-week-old mice were purchased from Harlan, Israel. All mice were kept in a specific pathogen-free facility. Mice were handled according to the criteria outlined in the "Guide for the Care and Use of Laboratory Animals" prepared by the National Academy of Sciences and published by the National Institutes of Health. All experiments were approved by the Animal Care Committee of the Hebrew University.

Tumor models

The mice were allowed to acclimate to the facility for at least one week prior to manipulation. Mice had free access to water and chow at all times. All animal procedures were performed under general anesthesia with intraperitoneally (i.p.) administered xylazine, 10 mg/g body weight (Chanelle Pharmaceuticals Manufacturing, Loughrea, Galway, Ireland) and ketamine, 450 mg/g body weight (Fort Dodge Animal Health, Fort Dodge, IA). After surgery, mice were allowed food and water *ad libitum*.

Subcutaneous tumors

Tumor xenografts were established by subcutaneous injection of log-phase growth viable cells, 10^7 (in 150 µL PBS) in case of Panc1 cells or 10^6 (in 100 µL PBS) in case of Panc-02 cells; the cells were injected into the flanks of the mice. When tumors reached an average volume of 80 mm^3, mice were divided into equal groups. LODERs were implanted into tumors under anesthesia. The tumor volume was calculated according to the following formula: $V = [\text{largest diameter} * \text{small diameter}^2]/2$

Intra-pancreatic orthotopic tumors

The mice were anesthetized; their abdomens were sterilized with alcohol (70%) and were positioned laterally. A small, left abdominal flank incision was made, and the pancreas tail with the spleen was carefully exposed under aseptic conditions. The tumor cells (10^6 cells/30µL PBS) were injected into the tail of pancreas using a 27G tuberculin syringe. After replacement of the pancreas into the abdominal cavity, the incision was closed in two layers using an absorbable surgical 6–0 vicryl suture for the peritoneum and a 4–0 vicryl suture for the skin. After surgery, mice were inspected daily. Tumor growth was followed by measurement of Luciferase levels. When the tumors were detected, mice were stratified and divided into treatment groups according to the Luciferase levels and treated as noted. For LODER™insertion, mice were anesthetized, the pancreas was exposed as described, and LODERs were attached to the tumor using a 7–0 vicryl suture. The abdominal cavities were closed as described. Pancreatic tumor growth was followed by Luciferase measurement twice a week.

Immunohistochemical staining

Immunohistochemistry was conductedon 5 µm thick formalin-fixed, paraffin-embedded tissue sections by standard procedures. Deparaffinization and rehydration

were followed by antigen retrieval using a pressure cooker with Glycine buffer (pH9) and CDC47. CDC47 primary antibodywas diluted 1:50 (Biocare Medical #CM137b from Pharmatrade). Secondary antibodies were from DAKO. Staining was developed with diamonobenzine using a kit from Zymed for H&E staining. TUNEL staining was performed using the In Situ Cell Death Detection Kit (Riche, cat# 11684795910). Slides were visualized using a Nikon microscope and analyzed using the Nis elements computer program (Nikon Instruments Inc.)

Priming

Priming (~first 1–2 days) – onset of drug release leads to apoptosis at the closest cell layers surrounding the drug delivery system, enabling more drug to penetrate further outwards. The tumor is still impervious to diffusion far from the closest layers.

To accelerate priming, LODER™ was designed to release a burst of 20% of its contents on the first day, which for aLODER™ containing a total drug load of 5 µg translates into 4×10^{13} molecules. Substituting a 1 mm^3 layer of volume into which the drug penetrates on the first day, which would contain 7.1×10^5 cells (as obtained from the [cell density]$^{3/2}$) in 'untreated' tumor, in Table 1), an average of 0.53×10^8 siG12D molecules per cell is expected. Therefore, even if the cell relative uptake is much smaller than the 0.21%, the lowest value in Table 2, the number of released molecules during that step will be sufficient to support immediate drug entrance into the cell layer at a depth of ~165 µm, allowing the priming step.

Statistical analysis

All data were subjected to statistical analysis using the Excel software package (Microsoft, Courtaboeuf, France). A two-tailed Student's t-test was used to determine the difference between the groups. Differences were considered significant at $P < 0.05$. Data are given as the mean ± SEM
